# Supplementary material for: Origami-inspired soft fluidic actuation for minimally invasive large-area electrocorticography
Source: Nat Commun. 2024 Jul 26;15:6290. doi: 10.1038/s41467-024-50597-2 (PMC11282215; doi:10.1038/s41467-024-50597-2)
Supplement: Supplementary file 1 — Supplementary Information [file 41467_2024_50597_MOESM1_ESM.pdf]

Supporting Information for

Origami-inspired soft fluidic actuation for minimally invasive large-area electrocorticography

Lawrence Coles,[a] Domenico Ventrella,[b] Alejandro Carnicer-Lombarte,[a] Alberto Elmi,[b] Joe G. Troughton,[a,c] Massimo Mariello,[c] Salim El Hadwe,[a,d] Ben J. Woodington,[a] Maria L. Bacci,[b] George G. Malliaras,[a] Damiano G. Barone,[a,d] Christopher M. Proctor *[a,c]

a Department of Engineering, University of Cambridge, Cambridge CB2 1PZ, United Kingdom

b Department of Veterinary Medical Sciences, Alma Mater Studiorum, Università di Bologna, Ozzano dell'Emilia 40064 (BO), Italy

c Institute of Biomedical Engineering, Engineering Science Department, University of Oxford, Oxford OX3 7DQ, UK

d Department of Clinical Neurosciences, University of Cambridge, Cambridge, CB2 0QQ, UK

Email: christopher.proctor@eng.ox.ac.uk

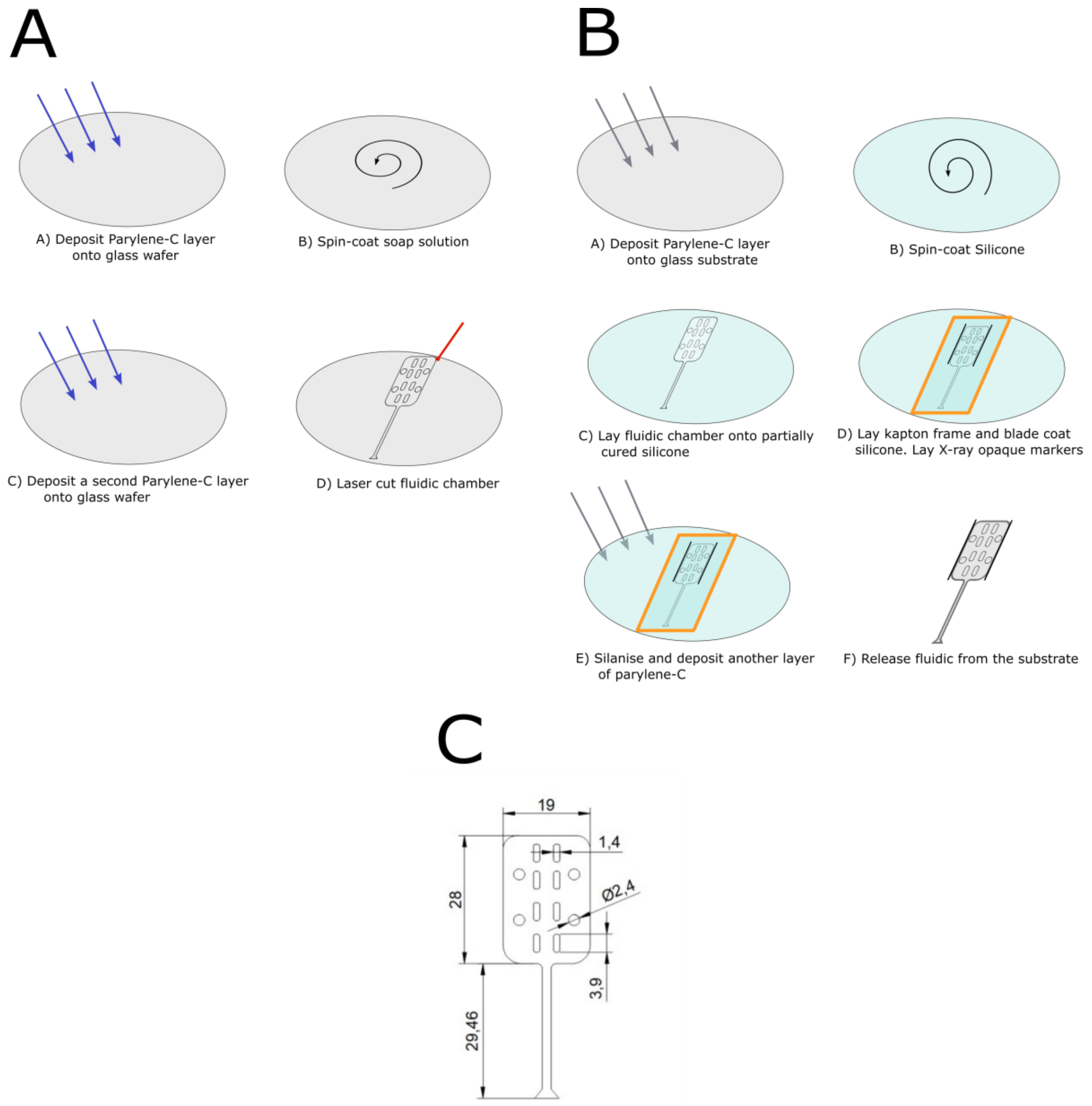

Fig. S1. Fabrication methodology of the fluidic design used in the MI-ECoG device. A) Fabrication steps for fabrication of the parylene-C fluidic chamber. B) Fabrication steps to form the full fluidic element of the MI-ECoG C) Design of the parylene-C fluidic chamber in the centre of the device. The laser cutting used to manufacture bonds the two layers of parylene-C to enable the chamber to withstand fluidic pressure. All dimensions are in mm.

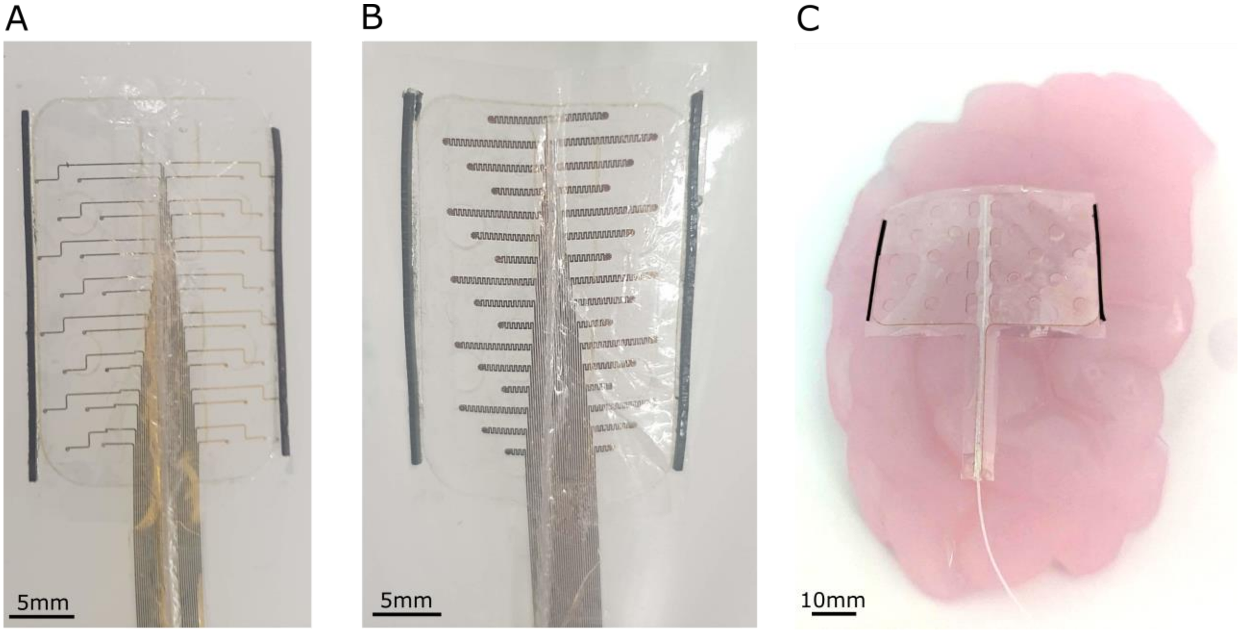

Fig. S2. Alternative MI-ECoG designs. A) MI-ECoG with a 32 gold electrode array with 0.3mm diameter microelectrodes. B) MI-ECoG with a 32 gold electrode array with 0.3mm diameter microelectrodes, with serpentine tracks integrated into the design for improved mechanical stability. C) Larger 50mm wide MI-ECoG fluidic placed on top of an agarose brain phantom. The flexibility of the MI-ECoG design platform is the ability of the geometry of the device to be tailored to the recording application, whilst maintaining the minimally invasive implantation footprint.

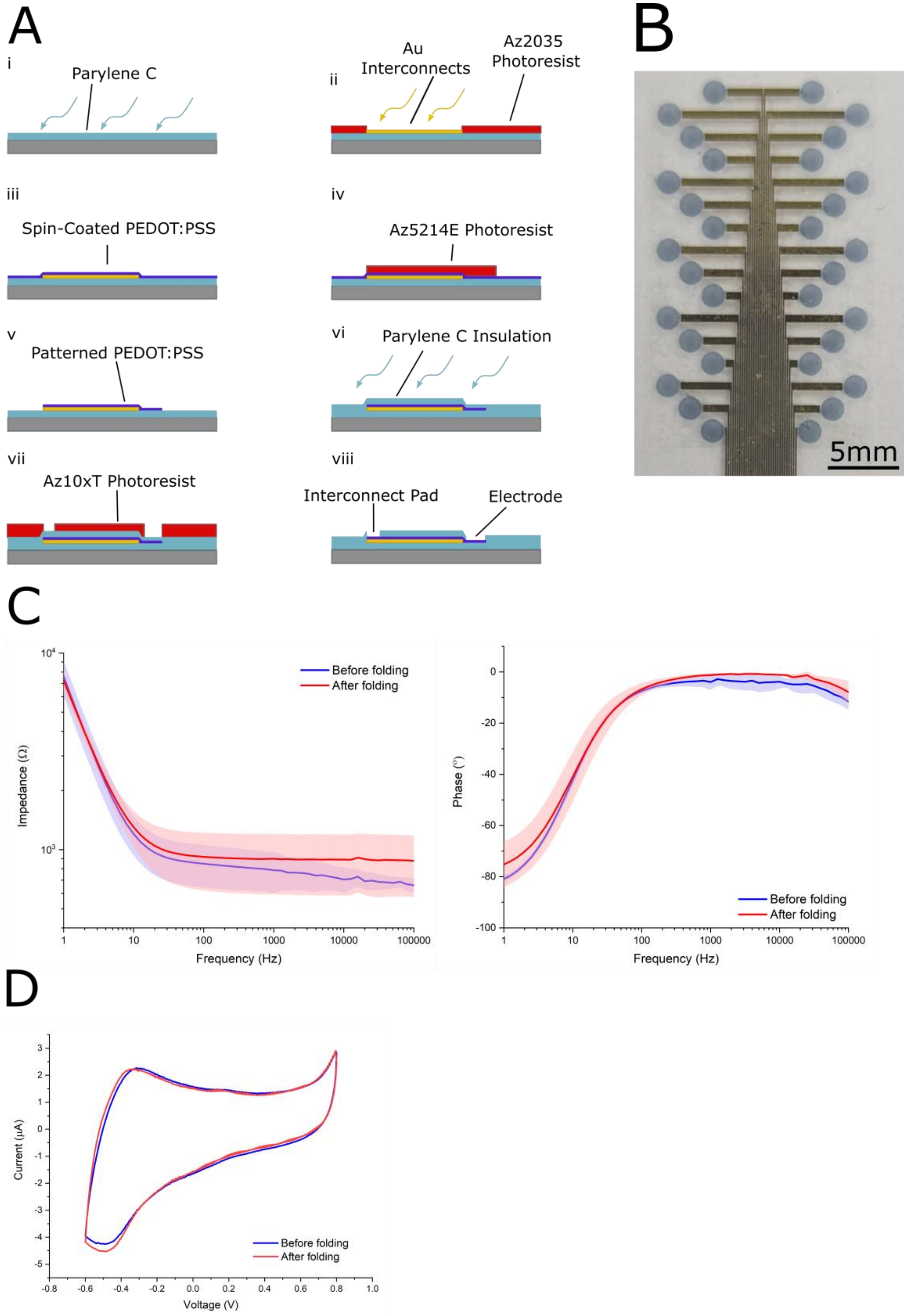
A
i
Parylene C
ii
Au Interconnects
Az2035 Photoresist
iii
Spin-Coated PEDOT:PSS
iv
Az5214E Photoresist
v
Patterned PEDOT:PSS
vi
Parylene C Insulation
vii
Az10xT Photoresist
viii
Interconnect Pad
Electrode
B
5mm
C
Impedance (Ω)
Frequency (Hz)
Before folding
After folding
Phase (°)
Frequency (Hz)
Before folding
After folding
D
Current (μA)
Voltage (V)
Before folding
After folding

Fig. S3. Design and characterisation of the PEDOT:PSS/Au electrode array. A) Overview of the fabrication steps to create the PEDOT:PSS electrodes and Au/PEDOT:PSS interconnects. i) 2µm Parylene C is deposited on a silicone wafer ii) Pattern photoresist and Au deposited with Ebeam evaporation iii) PEDOT:PSS spin-coated onto the wafer iv) Az5214E photoresist patterned on top of the PEDOT:PSS v) PEDOT:PSS layer patterned using reactive ion etching vi) 2µm Parylene C is deposited as the insulation layer vii) Az10xT photoresist patterned on to the Parylene C layers viii) Interconnect pad and electrode exposed with reactive ion etching. B) Image of the final fabricated electrode array, demonstrating the Au/PEDOT:PSS interconnects with the pure PEDOT:PSS electrodes. C) Frequency response analysis of the Au/PEDOT:PSS electrodes before and after folding, measured in PBS. When the PEDOT:PSS based electrode array was folded, there was a non-significant ($p = 0.2474$, two tailed t test) increase in mean electrode impedance from 1.05± 0.158 kΩ to 3.37 ± 9.9 kΩ. Data are presented as mean values +/- SD. D) Cyclic Voltammograms of the Au/PEDOT:PSS electrode before and after folding and packaging. The calculated cathodic Charge Storage Capacity has a non-significant ($p = 0.8371$, two tailed t test) change from 14.95 ± 3.87 mC/cm2 to 14.44 ± 1.29 mC/cm2.

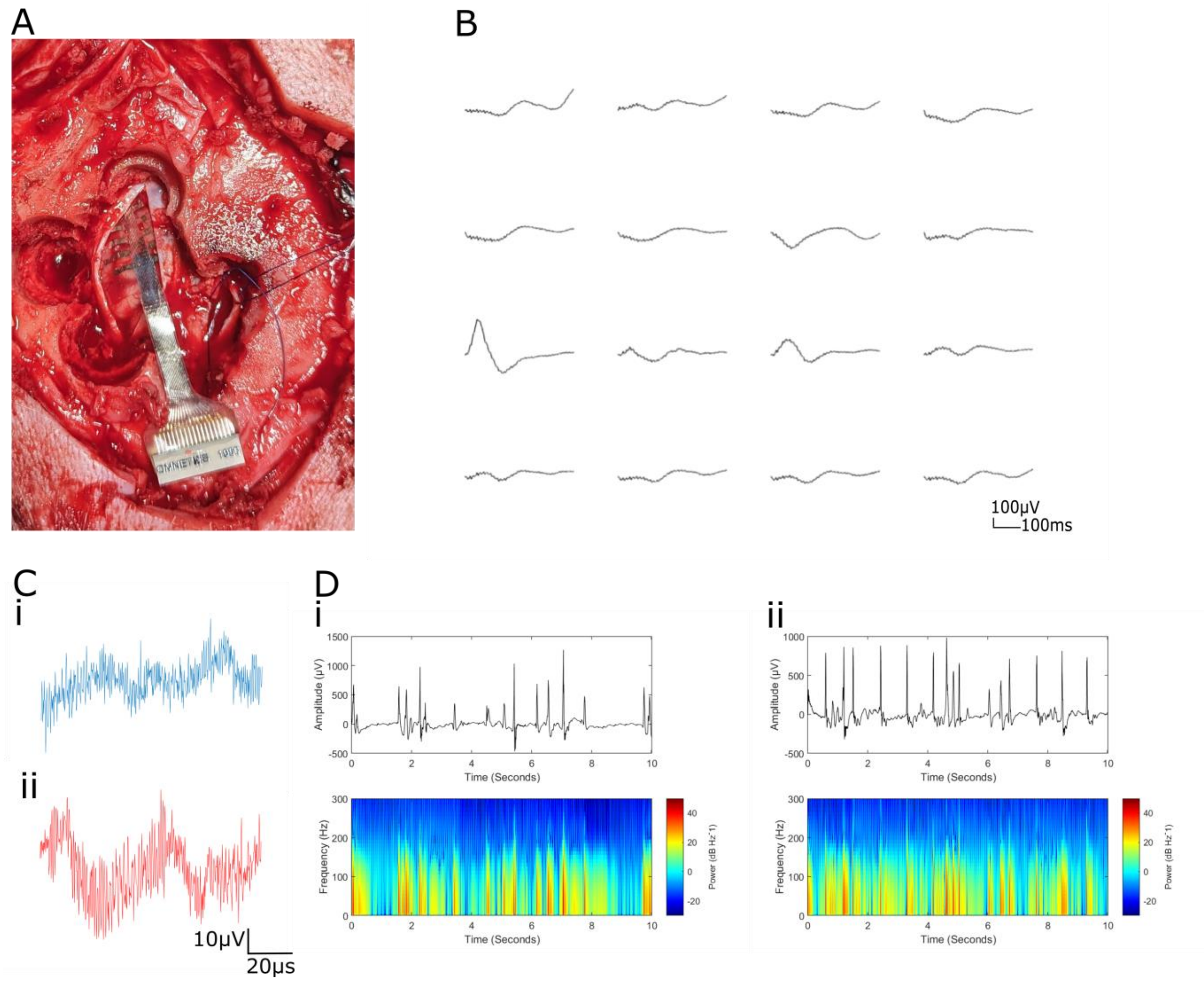

Fig. S4. Electrophysiology and comparison of the traditionally implanted, clinical PtIr ECoG array. A) Optical image of the PtIr array implanted through a full craniotomy and durotomy directly on the cortex. B) Recordings of induced AEPs) from the surface of the cortex from the implanted PtIr electrode array C) Noise floor profile of the AEP recordings obtained from the i) MI-ECoG and ii) PtIr ECoG. D) Raw traces and spectrogram showing AEPs being recorded by both the i) MI-ECoG and ii) PtIr ECoG. The two traces have a similar frequency profile within the recordings of the AEPs. Note that these traces are taken from different recording times in the same porcine model.

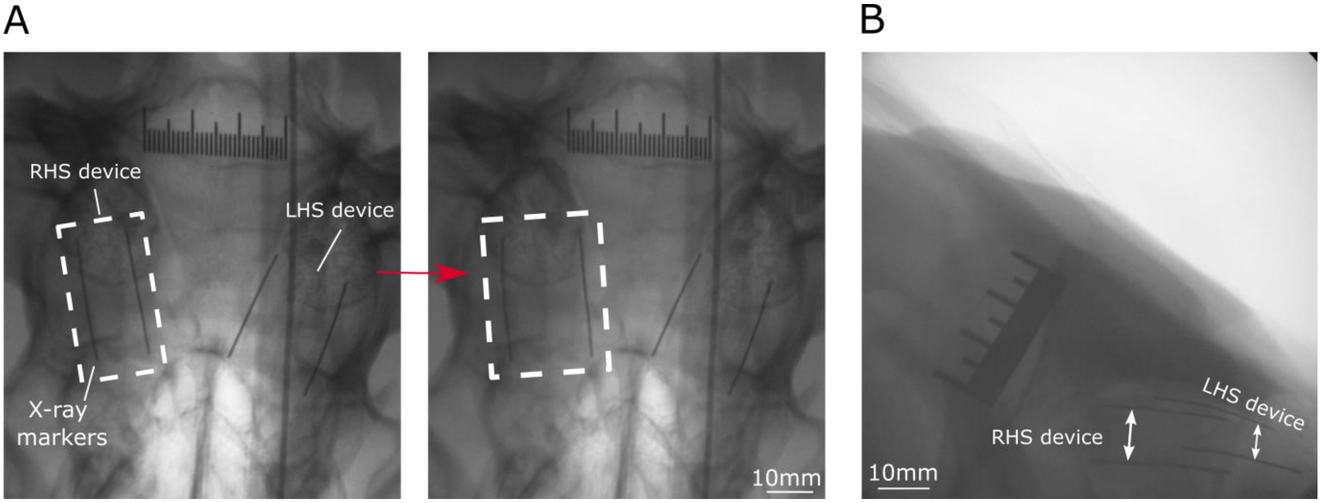

Fig. S5. Additional X-ray imaging of the expansion of a second MI-ECoG device subdurally onto the right-hand side (RHS) of the cortex. A) X-ray images of the MI-ECoG device before and after expansion of the device on the right-hand side of the cortex, with the white boxes showing the outline of the device tracked using the X-ray opaque markers. The implantation of the RHS MI-ECoG device followed the same implantation procedure described for the left-hand side (LHS) MI-ECoG device initially expanded. The LHS MI-ECoG device was left in place during the expansion of the second device. B) A lateral X-ray projection taken at a 15° angle showing the final position of both MI-ECoG devices after expansion

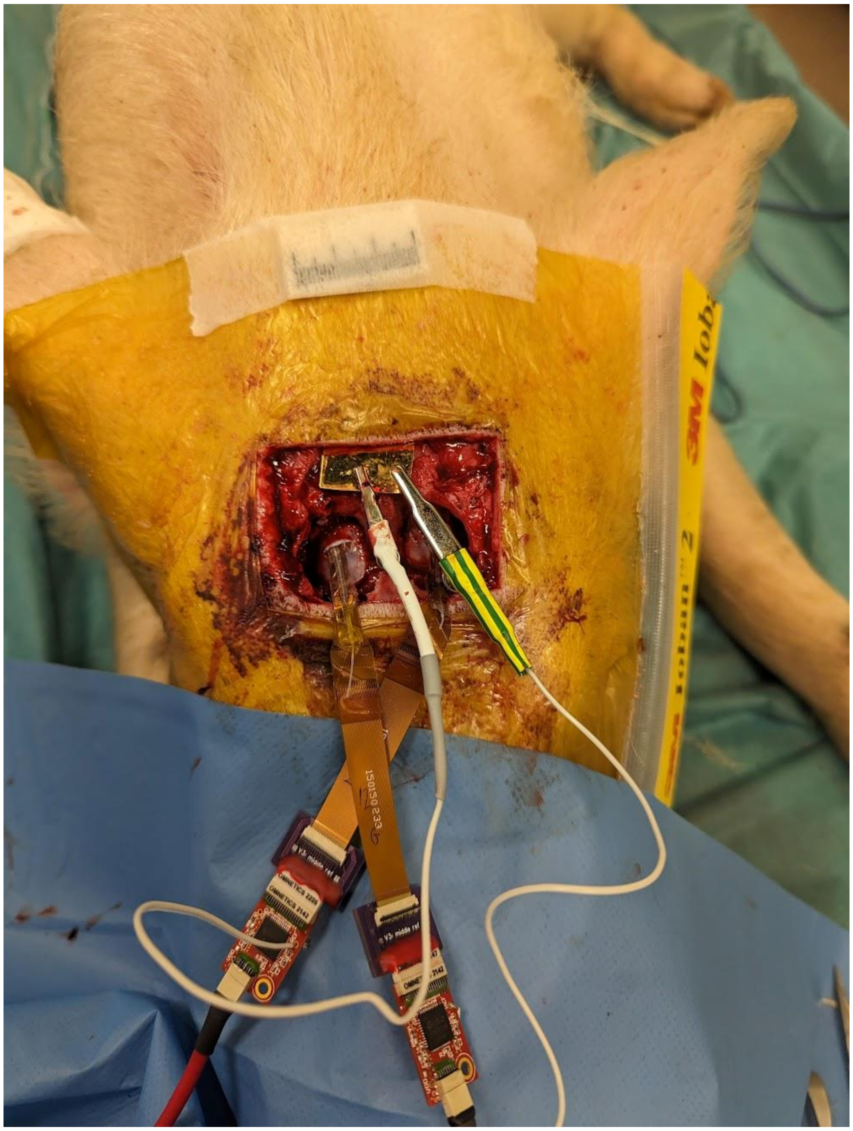

Fig. S6. Recording setup showing an MI-ECoG in the left burr-hole connected to an Intan 32-Channel RHS Stimulation/Recording head stage. The large gold reference electrode for the MI-ECoG is placed subcutaneously above the burr holes.

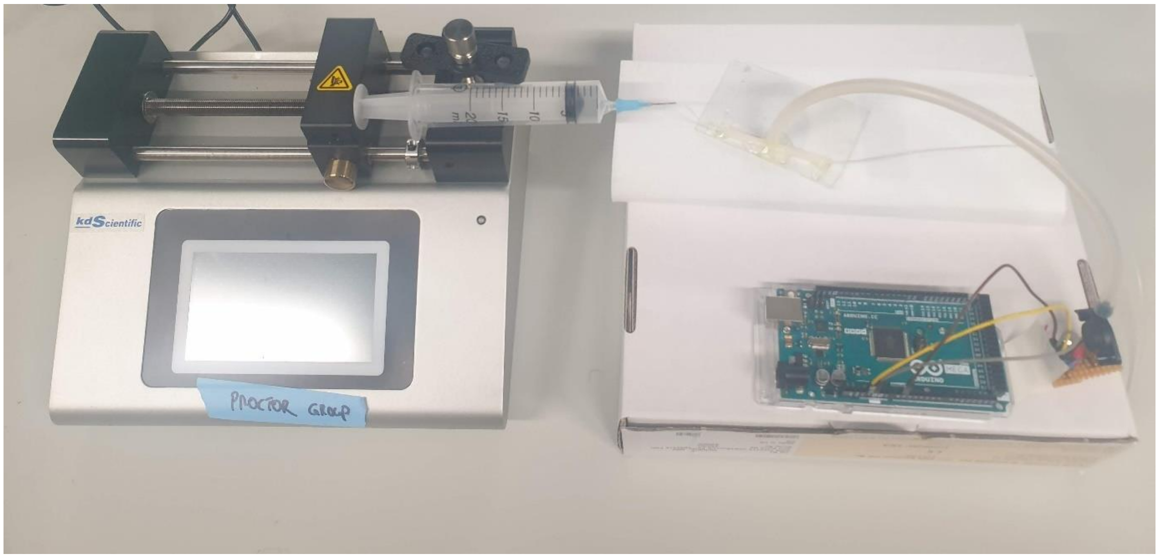

Fig. S7. The pressure testing setup used to record the pressure of the device during fluidic actuation. A 3D printed T connector is used to connect a pressure transducer, with the data recorded using an Arduino Mega. For maximum pressure testing, the syringe pump is used to inject either air or water at 1ml/min
